# Updated subnational estimates of Water, Sanitation and Hygiene access in Low- and Middle-Income countries: a spatially referenced hierarchical ordinal multinomial modeling analysis using R template model builder

**DOI:** 10.21203/rs.3.rs-9543036/v1

**Published:** 2026-04-28

**Authors:** Bin Fang, Nasif Hossain, Prakrut Kansara, Kelly Herdzik, Andrew Strumpf, Christian Damgaard, Vanessa Harris, Ben Zaitchik, Venkataraman Lakshmi, Margaret Kosek, Josh Colston

**Affiliations:** 1. Department of Civil and Environmental Engineering, University of Virginia, Charlottesville, Virginia, USA; 2. The Environmental Institute, University of Virginia, Charlottesville, Virginia, USA; 3. Department of Earth and Planetary Sciences, Johns Hopkins University, Baltimore, Maryland, USA; 4. Amsterdam Institute for Global Health & Development, Amsterdam, The Netherlands; 5. Research Computing, University of Virginia, Charlottesville, Virginia, USA; 6. Department of Ecoscience, Aarhus University, Denmark; 7. Amsterdam University Medical Centre, Amsterdam, The Netherlands; 8. Division of Infectious Disease and International Health, School of Medicine, University of Virginia, Charlottesville, Virginia, USA; 9. Department of Public Health Sciences, University of Virginia School of Medicine, Charlottesville, Virginia, USA

**Keywords:** Water, Sanitation, and Hygiene (WASH), Social Determinants of Health, Geostatistical modeling, Household surveys, Cross-sectional data, Global Health

## Abstract

Access to safe drinking water, improved sanitation, and basic hygiene is a critical factor of infectious disease risk and child health, particularly in low- and middle-income countries (LMICs). Spatially detailed information on household-level water, sanitation, and hygiene (WASH) conditions is critical for characterizing pathways of infectious disease transmission and exposure; however, such information is not directly available for most locations. We produced a harmonized global dataset of WASH conditions derived from 376 nationally representative household surveys, including the Demographic and Health Surveys (DHS), Multiple Indicator Cluster Surveys (MICS), and national surveys, covering more than six million households and approximately 291,000 georeferenced clusters across LMICs. Drinking water source, sanitation facility type, and hygiene status were classified as ordered categorical variables reflecting service levels. Household survey data were integrated with 24 environmental and socioeconomic covariates from multiple data sources. Spatial ordinal regression models were fit using R Template Model Builder (RTMB), incorporating cluster-level random effects and spatial random fields represented by the stochastic partial differential equation (SPDE) formulation. The resulting dataset provides high-resolution gridded estimates of WASH service levels and associated probabilities, suitable for geographic distribution pattern analyses, environmental health research, and public health planning.

## Background & Summary

Access to safe drinking water, improved sanitation, and basic hygiene facilities are crucial determinants of health associated with infectious disease transmission^1^, malnutrition^2^, gender-based violence^3^, and mortality.^4^ The effects of water, sanitation, and hygiene (WASH) access are most pronounced in low-and middle-income countries (LMICs), where 62.5% of pediatric diarrheal deaths are attributable to inadequate WASH.^5^ Transmission patterns of many pathogens, including enteric, respiratory, and soiltransmitted helminth infections, are strongly associated with the household WASH environment through multiple pathways.^1,6^ For example, unsafe water sources facilitate ingestion of microbial contaminants, poor sanitation enables environmental fecal contamination, and limited hygiene infrastructure constrains effective handwashing and household cleanliness. The WASH-associated burden of disease is particularly high in rural areas and regions across Africa, Asia, and Latin America, where infrastructure coverage remains incomplete and where climatic and environmental conditions may further increase pathogen persistence and exposure.^7^ Infants and young children are particularly vulnerable to WASH-associated pathogens, due to their high contact rates with contaminated environment and their reliance on household-level WASH conditions for protection against enteric infections.^8^ Despite efforts to achieve Sustainable Development Goal 6 to “ensure availability and sustainable management of water and sanitation for all” by 2030^9^, an estimated 2.1 billion people lack access to safe drinking water and 3.4 billion lack safely managed sanitation, with least developed countries bearing double the burden of high-income countries.^10^

While multilateral institutions like the WHO and UNICEF’s Joint Monitoring Programme track progress towards targets like SDG 6, the coverage data they release are aggregated to national level, or to strata like urban and rural areas, or wealth quintiles.^11^ Even within particular countries, WASH access remains highly heterogeneous, with sub-national and sub-regional inequalities that are masked by spatially aggregated statistics.^12^ Reliable estimates of WASH coverage at fine scale, global scope and derived by consistent methods are critical for identifying geographical hotspots of need and supporting integrated environmental health research.^12–14^ While in 2020, the Local Burden of Disease project investigators published geostatistically modelled sub-national estimates of various water source and sanitation facility categories for each single year, from 2000 to 2017^12^, these have not been kept updated, excluded numerous highly populous LMICs, were not spatially continuous across their target domain, and did not include a hygiene indicator.^15^ Other attempts to model WASH coverage across multiple countries have not gone below national level.^13,14^

In this study from the Planetary Child Health & Enterics Observatory (Plan-EO, www.planeo.earth^16^), we leverage recent improvements in geostatistical software and methods as well as the increased availability of harmonized survey and environmental data, to model coverage of each category of the three WASH component variables, and provide the stakeholder community with globally consistent, high-resolution datasets of the resulting predictions. We integrate data from a large number of nationally representative household surveys with environmental and socioeconomic predictors derived from multiple data sources. Furthermore, the WASH components are directly modeled as ordered categories within a spatial ordinal regression framework, allowing for up to five outcome categories, while also accounting for random effects from spatial autocorrelation and within-cluster non-independence. The resulting datasets provide a spatially consistent and detailed estimate of these key public health risk factors that preserve both ordinal structure and fine-scale geographic variability, with estimates produced at 5 km^2^ resolution. The production of accessible rasterized data contributes to a robust resource in support of public health planning, environmental epidemiology evaluation and prevention, and progress monitoring toward global WASH-related development targets.

## Methods

### Household survey data and outcome variables

**Figure 1:**
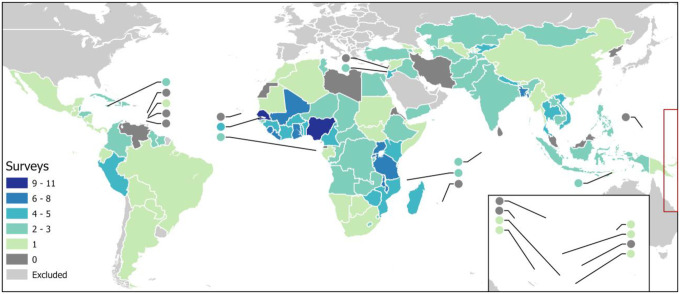
Number of nationally representative household surveys included in input dataset by country for included LMICs (small countries represented by circles).

We compiled data from 376 nationally representative household surveys conducted in LMICs since 2005 sourced from the Demographic and Health Surveys (DHS)^17^ and Multiple Indicator Cluster Surveys (MICS)^18^ programs, and, for countries where these were unavailable, additional surveys implemented by national governments.^19^ We included any surveys containing information on at least one of the WASH components from countries designated as LMICs by the World Bank’s definition as of 2020, excluding those fully located in Europe (Figure 1).^20^ All included surveys used multi-stage, cluster-randomized sampling designs, in which households are nested within clusters, typically census enumeration areas that serve as the primary sampling units, and clusters are further stratified by sub-national administrative region and urban/rural strata. In most surveys, approximately 25–30 households are sampled per cluster. Most DHS and some MICS survey datasets include displaced coordinates of the cluster locations.^21^ Where these were unavailable, we georeferenced clusters using a stratified, population-weighted approach that has been described previously.^19^ Combined, the resulting database contained information on WASH facilities for over six million households georefererenced to over 291,000 survey cluster locations. The three WASH components were each classified into categories reflecting increasing levels of service and infrastructure (i.e., from least to most improved service levels) and under the assumption that the categories constitute ordered bins of latent continuous variables (Table 1). We therefore chose reference categories for each WASH component based on conditions that pose the highest risk for enteric pathogen transmission. For water, unimproved surface water was used as the reference because these sources are most likely to be contaminated, especially during flooding, whereas tube wells are less likely to be contaminated by fecal matter during flooding events compared to taps, ponds, or other surface water sources.^22^ For sanitation, open defecation contributes directly to environmental fecal contamination. For hygiene, households with no observed handwashing facility served as the reference group since this represents the lowest level of protection against fecal-oral transmission (Table 1). Each of the three WASH components was modeled independently, generating separate class probabilities for water, sanitation, and hygiene. **Supplementary file 1** gives descriptive statistics about each category of each WASH component by survey, as well as the sources of each survey.

Covariate values for a suite of 24 environmental and socioeconomic variables available in raster format were extracted to each georeferenced cluster coordinate location.^19^ The covariates are listed in Table 2 and were selected based on their hypothesized associations with WASH access and infrastructure. Continuous covariates were standardized prior to model fitting to ensure numerical stability and comparability across predictors, while categorical variables (e.g., land cover type) were encoded as factor variables. In addition to these geographically varying covariates, time was modeled as the log-transformation of the number of days since January 1^st^, 2005.

### Ordinal Spatial Modeling Framework

Models were fitted to each of the three ordered categorical WASH component outcomes in turn using a spatially referenced hierarchical ordinal multinomial modeling framework implemented in the R Template Model Builder (RTMB) package.^23,24^ This framework links observed categorical outcomes to a latent continuous variable while accounting for spatial dependence and survey clustering. For observation *i*, the ordinal response *y*_*i*_ ∈ {1, … ,*K*} is assumed to arise from an underlying latent variable *z*_*i*_:

(1) $$z_{i}=\eta_{i}+u(s_{i})+b_{c(i)},$$

where $\eta_{i}={\text{x}}_{i}^{⊤}\beta$ is the fixed-effect linear predictor constructed from environmental and socioeconomic covariates, *u*(*s*_*i*_) is a spatial random field evaluated at location *s*_*i*_, and *b*_*c(i)*_ is a cluster-level random effect corresponding to survey cluster *c*. Observed outcome category is determined by a set of monotonically increasing threshold parameters *θ* = (*θ*_1_, …,*θ*_*K*−1_), as

(2) $$P(y_{i}\leq k)={\text{logit}}^{-1}(\theta_{k}-z_{i}),k=1,\ldots ,K-1.$$

This cumulative logit formulation preserves the ordinal structure of the response while allowing flexible incorporation of spatial and hierarchical effects. The fixed-effect component *η*_*i*_ includes environmental and socioeconomic predictors derived from remote sensing products and other data sources. These predictors were spatially aligned to survey data locations and standardized prior to model fitting to improve numerical stability in RTMB optimization and interpretability of coefficients.

Spatial autocorrelation was modeled using a Gaussian random field with Matérn covariance structure, represented through the Stochastic Partial Differential Equation (SPDE) approach.^25^ The SPDE formulation enables efficient approximation of a continuous spatial field over an irregular triangulated mesh constructed from survey cluster coordinates. The spatial field *u*(*s*) satisfies:

(3) $${\left(\kappa^{2}-\Delta \right)}^{\alpha /2}u(s)=W(s),$$

where *k* controls the spatial range, *α* determines field smoothness, Δ is the Laplacian operator, *u*(*s*) is a spatial random field, and *W*(*s*)is spatial white noise. The practical spatial range of the process is given by $\text{range}=\frac{\sqrt{8}}{\kappa }$, which represents the distance beyond which spatial correlation becomes negligible. The SPDE approach allows scalable inference for large datasets by projecting the spatial field onto a low-dimensional basis defined by the mesh nodes.

In contrast to previous Plan-EO risk factor modeling studies, we did not sample households within clusters. Instead, to account for within-cluster correlation occurring due to survey design and unobserved local heterogeneity, a cluster-level random effect *b*_*c(i)*_ was included. These effects were modeled as independent and identically distributed Gaussian random variables with mean zero and estimable variance. Inclusion of cluster effects helps separate local sampling variability from broader spatial patterns captured by the SPDE field.

### Model Estimation

Model parameters were estimated by maximizing the joint negative log-likelihood (JNLL), which combines contributions from the ordinal likelihood, cluster-level random effects, and spatial random field:

(4) $$\text{JNLL}=-\text{log}L_{\text{ordinal}}-\text{log}L_{\text{cluster}}-\text{log}L_{\text{SPDE}}$$

RTMB uses automatic differentiation and Laplace approximation to efficiently integrate over high-dimensional random effects, enabling robust estimation for very large numbers of household observations and spatial locations.

### Model Evaluation

The fitted models were used to generate high-resolution global prediction surfaces by evaluating the linear predictor and spatial field on a regular grid. Predicted class probabilities were derived from the cumulative logit formulation, enabling spatially explicit mapping of WASH service levels while preserving ordinal uncertainty. Prediction uncertainty was quantified by computing the standard errors (SE) of predicted class probabilities via the delta method on the linear predictor at each grid cell. These standard error maps highlight regions of lower confidence, typically corresponding to areas with sparse survey coverage or greater spatial variability. Model performance was evaluated using multiple diagnostic tools, including calibration curves, Dunn-Smyth residuals, and posterior predictive checks. Predictive accuracy was assessed using confusion-matrix-based metrics (area under the curve (AUC), precision, recall, F1 score, and accuracy) for each ordinal class.

## Data Record

The gridded predictions of WASH category prevalence in LMICs generated using the RTMB-based spatial ordinal regression framework are publicly available through the University of Virginia’s data repository (Dataverse). For each component, the dataset includes GeoTIFF raster layers representing the predicted probability of each ordinal class. In addition, standard error raster layers are provided to quantify prediction uncertainty, representing the standard error of the linear predictor at each grid cell. All products are provided at a nominal spatial resolution of ~5 km (0.05° grid spacing) on a global grid in the WGS84 geographic coordinate system (EPSG:4326), spanning 180° W to 180° E and 55° S to 55° N, consistent with the coverage of the input covariates and household survey data. Files are named following the format [component]prob[class].tif for predicted probabilities and [component]se[class].tif for corresponding standard error maps. The component indicates the WASH component (e.g., hygiene, sanitation, or water), and class corresponds to the associated ordinal category (e.g., Hwash_Bas, ImpS_Sew, ImpW_Pipe), as shown in Table 1 and the repository listing. A value for time corresponding to the first of January 2024 was used for making predictions.

### Data Overview

**Figure 2:**
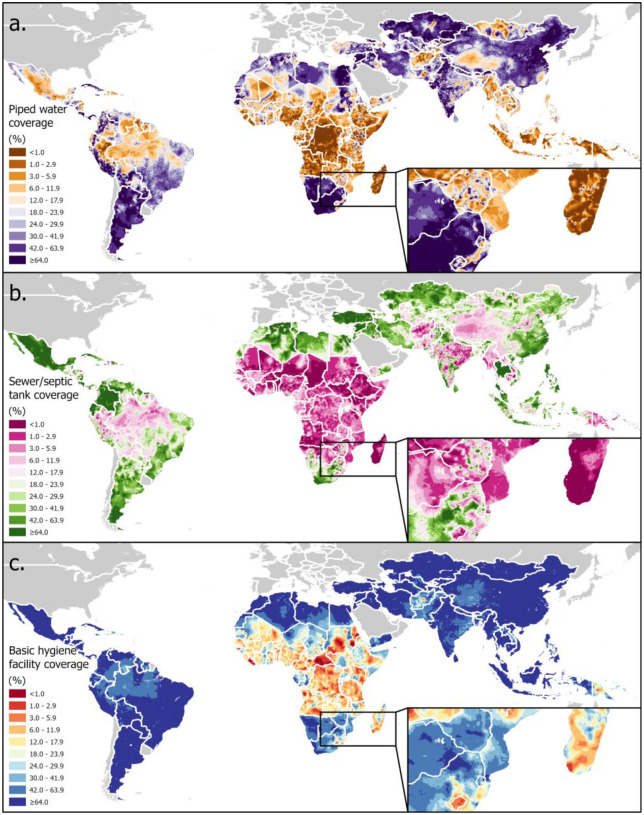
Predicted global probability maps for three classes of WASH components: piped water, sewer, and basic hygiene showing spatial patterns. Inset maps illustrate localized and detailed spatial variability of WASH components.

Figure 2 presents global maps of predicted probabilities for selected representative categories of water, sanitation, and hygiene components. These spatial predictions exhibit strong large-scale coherence, span a broad dynamic range and align with socioeconomic and environmental gradients in the model input predictors. Higher probabilities of improved service categories, including piped water supply, sewer-connected sanitation, and basic hygiene are observed in more urbanized and economically developed regions, with values approximately ranging from 0.6–0.9, while lower service levels prevail in much of Sub-Saharan Africa, South Asia, and other LMIC regions. Illustrated by the inset map panels outlined at the lower-right corner of each subplot, the prediction spatial pattern preserves localized and detailed heterogeneity at a finer spatial scale, which indicates the RTMB based model’s ability to capture sub-national spatial variability of the WASH components.

## Technical Validation

### Statistical diagnostics:

**Figure 3:**
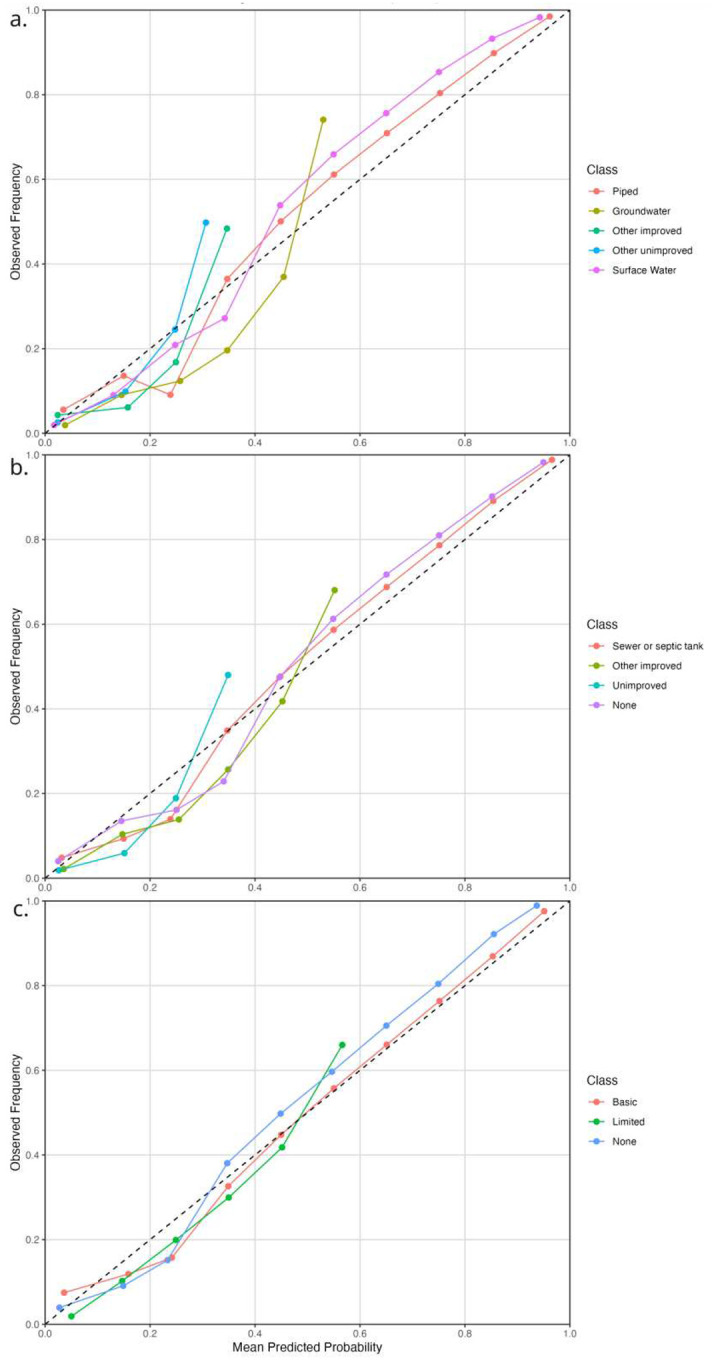
Probability calibration curve plots for a). Water source; b). Sanitation facility and; c). Hygiene facility.

**Figure 4:**
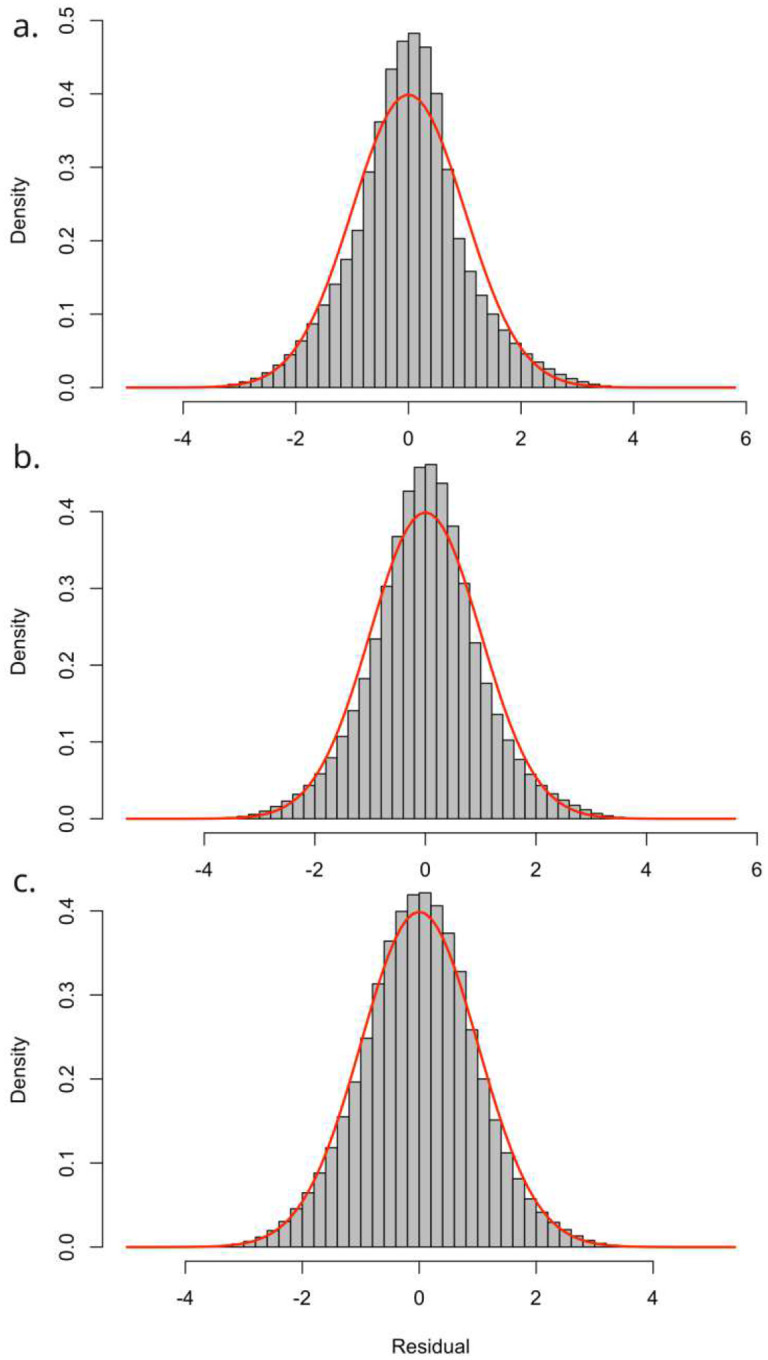
Histogram of Dunn-Smyth residuals for a). Water source; b). Sanitation facility and; c). Hygiene facility (theoretical standard normal density shown in red).

**Figure 5:**
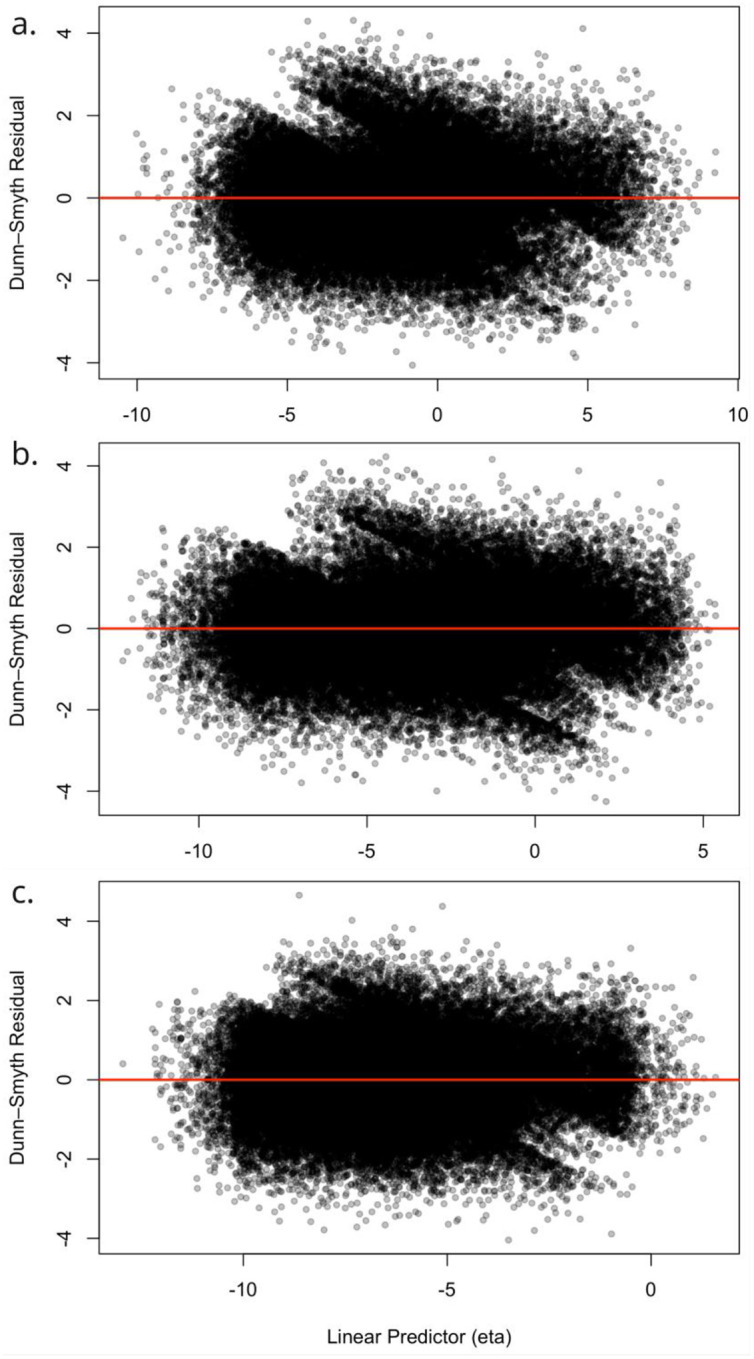
Scatter plots of Dunn-Smyth residuals against linear predictors from models for a). Water source; b). Sanitation facility, and; c). Hygiene facility.

**Figure 6:**
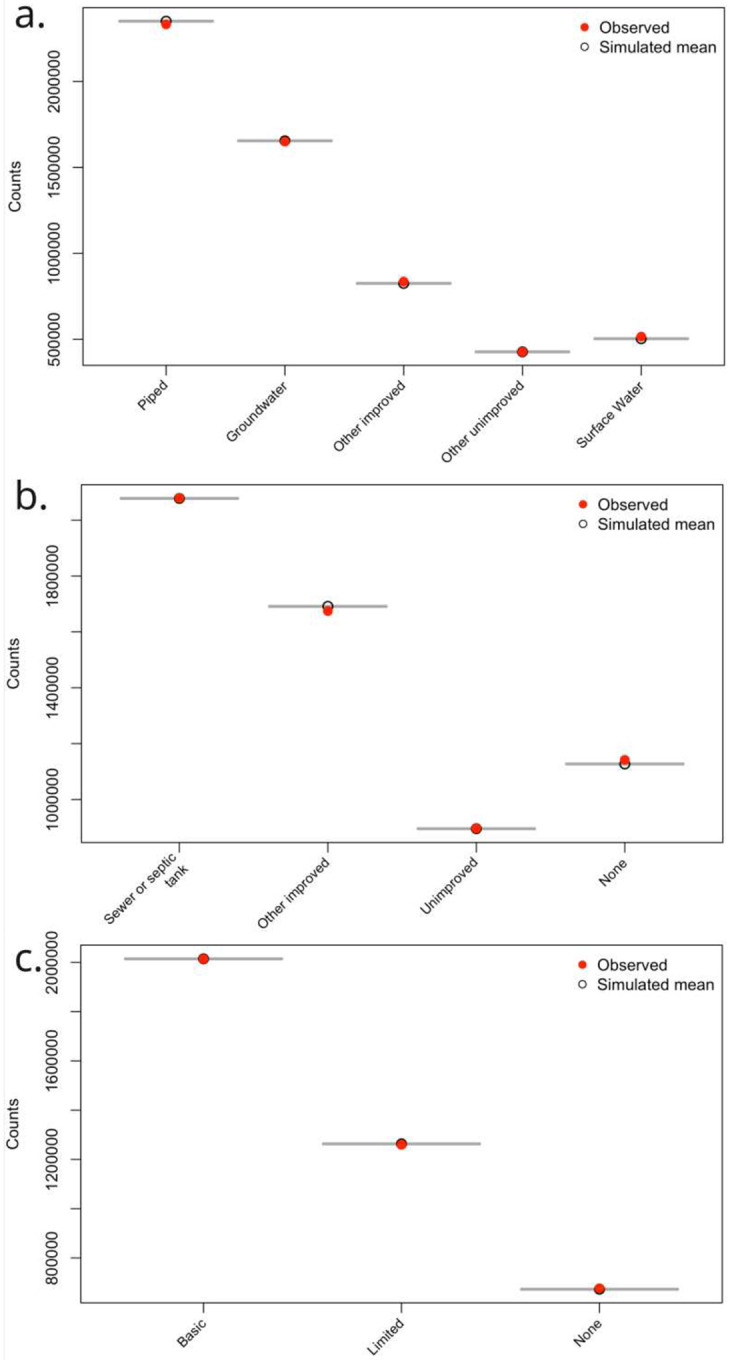
Posterior predictive checks for a). Water source; b). Sanitation facility, and; c). Hygiene facility.

The quality and reliability of the WASH prediction datasets were evaluated using a set of statistical metrics that included probabilistic calibration measures, residual-based model checks, and category-specific predictive performance metrics. These metrics were chosen to ensure that the resulting datasets are not only statistically well fitted, but also geospatially suitable for public health and environmental applications. Statistical diagnostics of model performance are presented in Figures 3–6 and they provide reliability of model adequacy. Probability calibration curves (Figure 3) compare predicted probabilities with observed outcome frequencies and show overall close agreement across most component categories, with deviation from the 1:1 line generally within 0.05–0.1 for common service categories, which indicates that the ordinal models are generally well calibrated. Modest under or over prediction is observed for less frequent categories but the discrepancy is overall less than 0.15, which is expected given the inherent category imbalance in large-scale household survey data. Overall, the results demonstrate that the models produce well-calibrated probabilistic outputs across WASH components.

Dunn–Smyth residuals (Figure 4) were examined to assess the distribution assumptions of the ordinal likelihood. The residuals exhibit approximately standard normal distribution with empirical means close to 0 and standard deviations near 1, and with minimal skewness and limited tail inflation, suggesting that the latent variable formulation and threshold structure provide an appropriate representation of the observed categorical responses. The red curve denotes the theoretical standard normal density, and the close agreement between this reference line and the histogram further indicates that the residuals conform well to the expected distribution under a correctly specified model. No strong spatial or systematic patterns were detected in the residuals, indicating that major features of spatial structure are effectively characterized by the model.

Figure 5 shows Dunn–Smyth residuals plotted against the linear predictor for each WASH component. Residuals are symmetrically distributed around zero with no noticeable trend or curvature, indicating that the linear predictor adequately captures the systematic variation in the data. Minor dispersion at extreme predictor values is observed but is limited and consistent with class imbalance in ordinal outcomes. These patterns indicate no major model misrepresentation and support the suitability of the link function and the inclusion of spatial and cluster-level random effects.

Posterior predictive checks (Figure 6) evaluate whether the fitted models reproduce key characteristics of the observed data. Simulated replicate datasets generated from the fitted models closely match the observed distributions of outcome categories, with differences typically below 5%, which supports the internal consistency of the modeling framework. In summary, these diagnostics demonstrate that the model provides a statistically coherent and reliable representation of the data-generating process.

### Class-specific predictive performance:

Quantitative predictive performance is summarized in Table 1 using confusion matrix based metrics for each WASH component. AUC values are consistently high across WASH components, ranging from approximately 0.83 to 0.93, while precision and recall values generally fall between 0.6 and 0.85 for well represented categories, indicating strong predictability of the model. On the other hand, less common or low-level service categories exhibit lower precision or recall (typically ranging 0.3–0.6), reflecting increased classification uncertainty. Despite class imbalance, overall accuracy remains high across all three components, ranging from 0.76 to 0.9 for improved service categories, indicating reliable population-level performance.

In summary, the spatial coherence of predicted surfaces, strong probabilistic calibration, well-behaved residual diagnostics, and robust class-specific performance metrics demonstrate the reliability of the generated datasets. The validation results indicate that the spatial ordinal RTMB model framework effectively captures both the large-scale geographic structure and uncertainty associated with the ordinal WASH outcome categories. Consequently, the datasets are suitable for applications in epidemiology, environmental health modeling, and policy-oriented analyses that require consistent, high-resolution representations of WASH conditions.

### Shapley Values:

**Figure 7:**
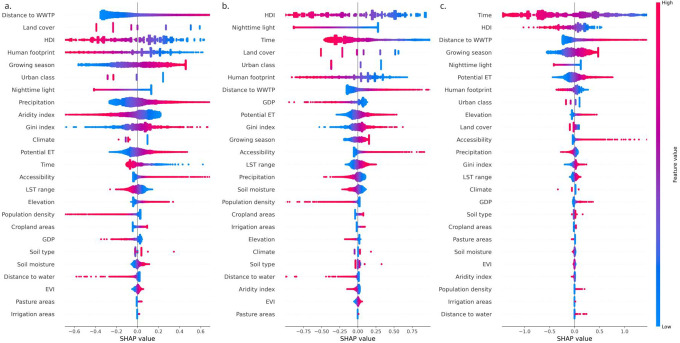
Shapley value plots of RTMB ordinal models for WASH variables: a) Water source; b). Sanitation facility; and c). Hygiene facility.

We assess the contribution of individual predictor variables to model outcomes using SHAP (SHapley Additive exPlanations) scores for three WASH indicators: (a) water source, (b) sanitation facility, and (c) handwashing facility (Figure 7). The SHAP summary plots depict the global importance of predictors, ranked from most to least influential from top to bottom. Each point corresponds to an individual observation, with its position along the x-axis indicating the SHAP value. Positive SHAP values indicate contributions toward higher predicted risk or lack of access, whereas negative values indicate reduced risk or increased access. The color gradient represents the magnitude of the predictor value, with red denoting high values and blue denoting low values. Across all three WASH indicators, socioeconomic variables dominate feature importance.

For water source (Figure 7a), model predictions are primarily driven by socioeconomic and topographical indicators, with distance to WWTP, landcover, HDI, and human footprint showing the strongest influence. High values (red) of these variables are consistently associated with negative SHAP values, indicating that greater development and urbanization reduce the predicted burden of inadequate water sources. For sanitation facilities (Figure 7b), HDI emerges as the dominant predictor, followed by nighttime light, time, and land cover. Higher HDI and nighttime light intensity are strongly associated with negative SHAP values, indicating improved sanitation access in more developed and electrified regions. The temporal variable time ranks highly (third), revealing a strong temporal trend in access, whereby later years are associated with negative SHAP values, reflecting progressive global improvements in sanitation infrastructure. For handwashing facilities (Figure 7c), predictions are primarily driven by temporal, socioeconomic, and environmental factors, with Time, HDI, distance to WWTP, growing season, and potential evapotranspiration ranking highest. Later years and higher HDI are associated with negative SHAP values, indicating progressive improvements and better hygiene access in more developed regions. Greater distance to WWTP, longer growing seasons, and higher potential evapotranspiration tend to produce positive SHAP values, suggesting a greater likelihood of limited hygiene access in infrastructure-poor, agriculturally intensive, or water-stressed regions.

## Usage Notes

The datasets accompanying this manuscript provide spatially explicit, model-based estimates of household-level WASH service levels across the LMICs. They are designed to support analyses of geographic variation in WASH access, investigation of environmental and socioeconomic relations of WASH conditions, and applications in infectious disease and child health research. The estimates were derived from a harmonized compilation of nationally representative household surveys, including DHS, MICS, and country-specific surveys. Models were fit using RTMB, incorporating cluster-level random effects and spatial random fields implemented through the SPDE approach. As a result, estimates reflect both observed data and spatial borrowing of strength across neighboring locations and may differ from national-level survey summaries or administrative statistics. The dataset includes gridded estimates of WASH service level probabilities and associated uncertainty measures. Users are strongly encouraged to account for uncertainty in downstream analyses, particularly when making comparisons across locations, time periods, or service categories where estimated differences may be small. Spatial estimates are aligned to the administrative boundaries and grid definitions provided with the data. When linking these outputs to external spatial or administrative datasets, users should verify boundary consistency and geographic identifiers, as administrative units and definitions may vary across sources and over time. Limitation: The classification of water service levels in this dataset does not explicitly incorporate certain dimensions of physical accessibility that have been considered in other studies. Studies consider whether the water source was >30 minutes away^11^ and whether it was piped onto the premises, or a communal location.^12^

## Supplementary Files

This is a list of supplementary files associated with this preprint. Click to download.

- WaSHSupplementaryfileS1data.xlsx

## Figures and Tables

**Table 1:** Outcome variables with ordinal categories, definitions based on typical survey responses, and names of prediction raster files

Variable	Ordinal ranking	Category	Definition^26^	File name
**Water source**	4	Improved: Piped	Water piped into dwelling, property, neighbor’s property, or to public tap or standpipe.	water_prob_ImpW_Pipe.tif water_se_ImpW_Pipe.tif
	3	Improved: Groundwater	Protected well, tube well or borehole.	water_prob_ImpW_Grd.tif water_se_ImpW_Grd.tif
	2	Improved: Other	Protected spring, rainwater, tanker truck, cart with small tank, bottled water etc.	water_prob_ImpW_Oth.tif water_se_ImpW_Oth.tif
	1	Unimproved: Other	Unprotected well, other sources.	water_prob_UnimpW_Oth.tif water_se_UnimpW_Oth.tif
	0: Ref.	Unimproved: Surface water	Unprotected spring, river, reservoir, lake, pond, stream, canal, irrigation channel.	water_prob_UnimpW_Srf.tif water_se_UnimpW_Srf.tif
**Sanitation facility**	3	Improved: Sewer or septic tank	Latrine with flush to a piped sewer system or septic tank.	sanitation_prob_ImpS_Sew.tif sanitation_se_ImpS_Sew.tif
	2	Improved: Other	Flush - to pit latrine, flush - don’t know where, pit latrine - ventilated improved pit (VIP), pit latrine - with slab, composting toilet.	sanitation_prob_ImpS_Oth.tif sanitation_se_ImpS_Oth.tif
	1	Unimproved: Other	Flush - to somewhere else, pit latrine - without slab / open pit, bucket toilet, hanging toilet/latrine.	sanitation_prob_UnimpS_San.tif sanitation_se_UnimpS_San.tif
	0: Ref.	Open defecation	No facility/bush/field.	sanitation_prob_Open_Def.tif sanitation_se_Open_Def.tif
**Hygiene facility**	2	Basic	Handwashing facility (fixed or mobile) with soap and water observed.	hygiene_prob_Hwash_Bas.tif hygiene_se_Hwash_Bas.tif
	1	Limited	Any handwashing facility (fixed or mobile) observed but without soap or water.	hygiene_prob_Hwash_Lim.tif hygiene_se_Hwash_Lim.tif
	0: Ref.	None	No handwashing facility of any kind observed.	hygiene_prob_Hwash_None.tif hygiene_se_Hwash_None.tif

**Table 2:** Definitions and sources of variables included as covariate predictors in the model

Variable	Definition	Units/Categories^1^	Source
**Accessibility to cities**	Travel time to nearest settlement of >50,000 inhabitants.	Minutes	MAP^27^
**Aridity index**	Mean annual precipitation / Mean annual reference evapotranspiration, 1970–2000.	Ratio	CGIAR-CSI^28^
**Climate zone**	First level Köppen-Geiger climate classification.	Tropical; arid; temperate; cold; polar	Beck et al. 2018^29^
**Cropland areas**	Proportion of land given over to cropland, 2000.	Proportion	CIESIN^30^
**Distance to major river**	Distance to major perennial river (derived from rivers and lakes centerlines database).	Decimal degrees	Natural Earth^31^
**Distance to wastewater treatment plant**	Distance to an operational wastewater treatment plant (WWTP, derived from coordinates)	Decimal degrees	Hydro-WASTE^32^
**Elevation**	Elevation above sea level.	Meters	NOAA^33^
**Economic development**	Sub-national unit-level Gross Domestic Production (GDP) per capita, 2015	Constant 2011 int. USD	Kummu et al. 2018^34^
**Enhanced Vegetation Index**	Vegetation greenness corrected for atmospheric conditions and canopy background noise.	Ratio	USGS^35^
**Growing season length**	Reference length of annual agricultural growing period (baseline period 1961–1990).	Days	FAO, IIASA^36^
**Human development**	Sub-national unit-level Human Development Index (HDI), 2015	Scale from 0 to 1	Kummu et al. 2018^34^
**Human Footprint Index**	Human Influence Index (HII) normalized by biome and realm.	Percentage	CIESIN^37^
**Income inequality**	Sub-national unit-level Gini coefficient, 2023	Scale from 0 to 1	Chrisendo et al. 2025^38^
**Irrigated areas**	Percentage of land equipped for irrigation around the year, 2020.	Percentage	FAO^39^
**Land cover and use**	General class of vegetation, tree, and ice cover or purpose of land use, 2020 (resampled and reclassified from Global Land Cover and Land Use)	Built up; cropland; desert; semi-arid; short vegetation; snow or ice; tree cover; wetland	GLAD^40^
**Land Surface Temperature**	Interannual averages of daily land surface temperature estimates for daytime, nighttime, and day/nighttime range, 2003–2020.	K	MOD21A1N v006^41,42^
**Nighttime light**	The surface upward radiance from artificial light emissions extracted from at-sensor nighttime radiances at top-ofatmosphere.	nWatts·cm^−2^·sr^−1^	NASA Black Marble^43^
**Pasture areas**	Proportion of land given over to pasture, 2000.	Proportion	CIESIN^30^
**Population density**	Human population density per 1km2.	Inhabitants per km2	WorldPop^44^
**Potential evapotranspiration**	8-day sum of the water vapor flux under ideal conditions of complete ground cover by plants.	kg/m^2^/8-day	NASA EOSDIS^45^
**Soil type**	Texture or classification of soil used to predict water holding capacity.	Coarse; Medium; Medium fine; Fine; Very fine; Organic; Tropical organic.	ECMWF Integrated Forecast System^46,47^
**Soil moisture**	Water content of soil	m^3^/m^3^	Fang et al. 2025^48^
**Urbanicity**	Urbanicity status at georeferenced location (reclassified from Global Human Settlement database).	Urban; peri-urban; rural; remote	GHS^49^

**Table 3:** Confusion-matrix-based performance metrics for the models of water source, sanitation facility, and hygiene facility.

Component	Class	AUC	Precision	Recall	F1	Accuracy	Support
**Water**	**Improved - Groundwater**	0.89	0.63	0.81	0.70	0.81	1,650,539
	**Improved - Other**	0.83	0.40	0.74	0.51	0.79	835,910
	**Improved - Piped Water**	0.93	0.85	0.86	0.85	0.88	2,332,610
	**Unimproved - Other**	0.84	0.18	0.84	0.29	0.70	427,151
	**Unimproved – Surface Water**	0.92	0.46	0.80	0.59	0.90	515,715
**Sanitation**	**Improved - Other**	0.86	0.58	0.82	0.68	0.78	1,674,514
	**Improved - Sewer**	0.93	0.82	0.86	0.84	0.88	2,078,974
	**Unimproved Sanitation**	0.87	0.40	0.84	0.54	0.78	896,739
	**Open Defecation**	0.90	0.69	0.73	0.71	0.88	1,141,983
**Hygiene**	**Basic facility**	0.90	0.80	0.83	0.82	0.82	2,013,747
	**Limited facility**	0.86	0.60	0.81	0.69	0.76	1,259,511
	**No facility**	0.91	0.67	0.80	0.72	0.89	677,011

## Data Availability

The dataset is publicly available through the University of Virginia’s Dataverse repository at: https://doi.org/10.18130/V3/O58DEE.

## Code Availability

The WASH component predicted map dataset was produced using open-source software package R. The relevant code files are publicly available at: https://doi.org/10.18130/V3/ED1WNW.

## References

[1] Wolf J. Burden of disease attributable to unsafe drinking water, sanitation, and hygiene in domestic settings: a global analysis for selected adverse health outcomes. The Lancet 401, 2060–2071 (2023). 10.1016/S0140-6736(23)00458-0 PMC10290941 37290458

[2] Anyanwu O., Ghosh S., Kershaw M., Cherinet A. & Kennedy E. Dietary Outcomes, Nutritional Status, and Household Water, Sanitation, and Hygiene (WASH) Practices. Curr. Dev. Nutr. 6, nzac020 (2022). 10.1093/cdn/nzac020 PMC8982029 35391902

[3] Pommells M., Schuster-Wallace C., Watt S. & Mulawa Z. Gender Violence as a Water, Sanitation, and Hygiene Risk: Uncovering Violence Against Women and Girls as It Pertains to Poor WaSH Access. Violence Women 24, 1851–1862 (2018). 10.1177/1077801218754410 29546802

[4] Andres L. A., Borja-Vega C., Fenwick C., de Jesus Filho J. & Gomez-Suarez R. Overview and meta-analysis of global water, sanitation, and hygiene (WASH) impact evaluations. World Bank Policy Res. Work. Pap. (2018).

[5] Pru ss-Ustu n A. Burden of disease from inadequate water, sanitation and hygiene for selected adverse health outcomes: An updated analysis with a focus on low- and middle-income countries. Int. J. Hyg. Environ. Health 222, 765–777 (2019). 31088724 10.1016/j.ijheh.2019.05.004 PMC6593152

[6] Colston J. M. Associations between Household-Level Exposures and All-Cause Diarrhea and Pathogen-Specific Enteric Infections in Children Enrolled in Five Sentinel Surveillance Studies. Int. J. Environ. Res. Public. Health 17, 8078 (2020). 33147841 10.3390/ijerph17218078 PMC7663028

[7] Wu Z., Xia F. & Lin R. Global Burden of Diarrheal Diseases Linked to WaSH, 1990–2021: A Systematic Analysis of the Global Burden of Disease Study 2021. ACS EST Water 5, 3870–3878 (2025).

[8] Sly J. L. & Carpenter D. O. Special vulnerability of children to environmental exposures. Rev. Environ. Health 27, (2012). 10.1515/reveh-2012-0024 23095179

[9] SDG Fund. Sustainable development goals. Available This Link https://www.un.org/sustainabledevelopment/inequality/ (2015).

[10] World Health Organization & United Nations Children’s Fund. Progress on Household Drinking Water, Sanitation and Hygiene 2000–2024: Special Focus on Inequalities. (World Health Organization, 2025).

[11] World Health Organization & UNICEF. JMP Methodology: 2017 update and SDG baselines. https://washdata.org/reports/jmp-2017-methodology.

[12] Deshpande A. Mapping geographical inequalities in access to drinking water and sanitation facilities in low-income and middle-income countries, 2000–17. Lancet Glob. Health 8, e1162–e1185 (2020). 32827479 10.1016/S2214-109X(20)30278-3 PMC7443708

[13] Swe K. T. Impact of poverty reduction on access to water and sanitation in low- and lower-middle-income countries: country-specific Bayesian projections to 2030. Trop. Med. Int. Health 26, 760–774 (2021). 33813768 10.1111/tmi.13580

[14] Rahut D. B., Singh A. & Sonobe T. WASH facilities prevalence and determinants: Evidence from 42 developing countries. Front. Environ. Sci. 10, (2022).

[15] Institute for Health Metrics and Evaluation. Water, Sanitation, and Hygiene. VizHub https://vizhub.healthdata.org/lbd/wash (2026).

[16] Colston J. M. The Planetary Child Health & Enterics Observatory (Plan-EO): A protocol for an interdisciplinary research initiative and web-based dashboard for mapping enteric infectious diseases and their risk factors and interventions in LMICs. PLOS ONE 19, e0297775 (2024). 38412156 10.1371/journal.pone.0297775 PMC10898779

[17] ICF International. Demographic and Health Surveys (various, 2000–2025). ICF https://www.icf.com/clients/health/survey-national-health-data-collection (2025).

[18] UNICEF. Multiple Indicator Cluster Survey (MICS). https://mics.unicef.org/surveys (2014).

[19] Colston J. M. Spatial variation in housing construction material in low- and middle-income countries: A Bayesian spatial prediction model of a key infectious diseases risk factor and social determinant of health. PLOS Glob. Public Health 4, e0003338 (2024). 39693286 10.1371/journal.pgph.0003338 PMC11654929

[20] The World Bank. The world by region. https://datatopics.worldbank.org/sdgatlas/archive/2017/the-world-by-region.html (2017).

[21] Burgert C., Colston J., Roy T. & Zachary B. Geographic Displacement Procedure and Georeferenced Data Release Policy for the Demographic and Health Surveys. http://dhsprogram.com/publications/publication-SAR7-Spatial-Analysis-Reports.cfm (2013).

[22] Martinez P. P. Tube Well Use as Protection Against Rotavirus Infection During the Monsoons in an Urban Setting. J. Infect. Dis. 221, 238–242 (2020). 31776559 10.1093/infdis/jiz436 PMC6936003

[23] Kristensen K., Nielsen A., Berg C. W., Skaug H. & Bell B. M. TMB: Automatic Differentiation and Laplace Approximation. J. Stat. Softw. 70, 1–21 (2016).

[24] Kristensen K. RTMB: ‘R’ Bindings for ‘TMB’. (2026).

[25] Lindgren F., Seaton A., Suen M. H. & Bachl F. E. fmesher: Triangle Meshes and Related Geometry Tools. (2026).

[26] Croft T. N., Allen C. K. & Zachary B. W. Guide to DHS Statistics (2023).

[27] Weiss D. J. A global map of travel time to cities to assess inequalities in accessibility in 2015. Nature 553, 333–336 (2018). 29320477 10.1038/nature25181

[28] Trabucco A. & Zomer R., J. Global Aridity Index and Potential Evapo-Transpiration (ET0). https://cgiarcsi.comunity (2018).

[29] Beck H. E. Present and future Ko ppen-Geiger climate classification maps at 1km resolution. Sci. Data 5, 180214 (2018). 30375988 10.1038/sdata.2018.214 PMC6207062

[30] Ramankutty N., Evan A. T., Monfreda C. & Foley J. A. Farming the planet: 1. Geographic distribution of global agricultural lands in the year 2000. Glob. Biogeochem. Cycles 22, n/a–n/a (2008).

[31] Natural Earth. Rivers and Lakes Centerlines 4.1.0. (2021).

[32] Ehalt Macedo H. Distribution and characteristics of wastewater treatment plants within the global river network. Earth Syst. Sci. Data 14, 559–577 (2022).

[33] Hastings D. A. & Dunbar P. K. Global Land One-Kilometer Base Elevation (GLOBE) Digital Elevation Model, Documentation, Volume 1.0. (1999).

[34] Kummu M., Taka M. & Guillaume J. H. A. Gridded global datasets for Gross Domestic Product and Human Development Index over 1990–2015. Sci. Data 5, 180004 (2018). 29406518 10.1038/sdata.2018.4 PMC5800392

[35] U.S. Geological Survey. Landsat Enhanced Vegetation Index. Landsat Missions https://www.usgs.gov/landsat-missions/landsat-enhanced-vegetation-index (2021).

[36] The Food and Agriculture Organization (FAO) & International Institute of Applied Systems Analysis. Global Agro-ecological Zones (GAEZ v3.0). (2012).

[37] Wildlife Conservation Society & Center for International Earth Science Information Network - CIESIN. Last of the Wild Project, Version 2, 2005 (LWP-2): Global Human Footprint Dataset (Geographic). (2005).

[38] Chrisendo D. Rising income inequality across half of global population and socioecological implications. Nat. Sustain. 8, 1601–1613 (2025).

[39] Mehta P. Half of twenty-first century global irrigation expansion has been in water-stressed regions. Nat. Water 2, 254–261 (2024).

[40] Potapov P. The Global 2000–2020 Land Cover and Land Use Change Dataset Derived From the Landsat Archive: First Results. Front. Remote Sens. 3, (2022).

[41] Hulley Glynn & Hook Simon. MOD21A1N MODIS/Terra Land Surface Temperature/3-Band Emissivity Daily L3 Global 1km SIN Grid Night V006. NASA EOSDIS Land Processes DAAC 10.5067/MODIS/MOD21A1N.006 (2017).

[42] Hulley Glynn & Hook Simon. MOD21A1D MODIS/Terra Land Surface Temperature/3-Band Emissivity Daily L3 Global 1km SIN Grid Day V006. NASA EOSDIS Land Processes DAAC 10.5067/MODIS/MOD21A1D.006 (2017).

[43] Roma n M. O. NASA’s Black Marble nighttime lights product suite. Remote Sens. Environ. 210, 113–143 (2018).

[44] Tatem A. J. WorldPop, open data for spatial demography. Sci. Data 4, 170004 (2017). 28140397 10.1038/sdata.2017.4 PMC5283060

[45] Running Steve, Mu Qiaozhen & Zhao Maosheng. MOD16A2 MODIS/Terra Net Evapotranspiration 8-Day L4 Global 500m SIN Grid V006. NASA EOSDIS Land Processes DAAC 10.5067/MODIS/MOD16A2.006 (2017).

[46] Balsamo G. A Revised Hydrology for the ECMWF Model: Verification from Field Site to Terrestrial Water Storage and Impact in the Integrated Forecast System. https://doi.org/10.1175/2008JHM1068.1 (2009) doi:10.1175/2008JHM1068.1.

[47] Mun oz-Sabater J. ERA5-Land: a state-of-the-art global reanalysis dataset for land applications. Earth Syst. Sci. Data 13, 4349–4383 (2021).

[48] Fang B., Lakshmi V., Hain C. & Mishra V. A global 400-m high-resolution soil moisture dataset derived from multi-sensor remote sensing observations. Sci. Data 13, 65 (2025). 41392295 10.1038/s41597-025-06356-z PMC12820223

[49] Pesaresi M. Operating Procedure for the Production of the Global Human Settlement Layer from Landsat Data of the Epochs 1975, 1990, 2000, and 2014 | EU Science Hub. https://ec.europa.eu/jrc/en/publication/operating-procedure-production-global-human-settlement-layer-landsat-data-epochs-1975-1990-2000-and (2016).

